# Beyond Gastroenteritis: A Case of Salmonella Infection Leading to Adrenal Insufficiency

**DOI:** 10.7759/cureus.94443

**Published:** 2025-10-13

**Authors:** Thwe Thwe Wint Htal, Aye Pyae Tin Hla, Aye Moh Moh Paing, Kyaw Thura, Clare Pollard

**Affiliations:** 1 Intensive Care Unit, Glan Clwyd Hospital, Bodelwyddan, GBR; 2 Medicine, Victoria Hospital Kirkcaldy, Kirkcaldy, GBR; 3 Acute Medicine, West Middlesex University Hospital, Isleworth, GBR; 4 Acute Medicine, Warwick Hospital, South Warwickshire University NHS Foundation Trust, Isleworth, GBR

**Keywords:** addison's disease, adrenal crisis, adrenal gland abscess, bacterial infectious disease, disseminated salmonella, gastro enteritis, infection spread, primary adrenal insufficiency, tachycardia induced cardiomyopathy, travel-related infection

## Abstract

Primary adrenal insufficiency (PAI), or Addison’s disease, is a rare and potentially life-threatening condition that requires prompt diagnosis and management. While most cases are autoimmune, other causes include infections such as *Salmonella*, though this is rarely reported. We describe a case of bilateral adrenal abscesses and adrenal insufficiency secondary to disseminated *Salmonella* Saintpaul infection in a previously healthy 20-year-old male following travel to Thailand. This case highlights the importance of early recognition of adrenal involvement in septic patients with persistent fever and electrolyte imbalances, even in immunocompetent individuals.

## Introduction

Adrenal insufficiency is a condition in which the adrenal glands fail to produce adequate amounts of steroid hormones, primarily cortisol and aldosterone, leading to impaired stress response and electrolyte imbalances.

Primary adrenal insufficiency is most often caused by autoimmune destruction of the adrenal cortex. However, infectious aetiologies, including tuberculosis and bacterial sepsis, can also cause adrenal damage through haemorrhage or abscess formation. *Salmonella* infection, though typically self-limiting, may progress to bacteraemia and deep-seated abscesses in rare cases.

Non-typhoidal *Salmonella*, which includes the Saintpaul strain, is one of the most common sources of bacterial foodborne disease worldwide [1]. It has been documented that in Thailand, the most common serovars of *Salmonella* spp. isolated from humans were *Salmonella* Group B, including *Salmonella* Typhimurium, *Salmonella* Derby, *Salmonella* Anatum, and *Salmonella* Stanley. *Salmonella* Saintpaul was found in minced pork in the northern region of Thailand [2]. Dissemination of *Salmonella* to extraintestinal sites is uncommon, particularly in immunocompetent hosts. Here, we present a rare case of bilateral adrenal abscesses and adrenal insufficiency in an immunocompetent young adult due to disseminated *Salmonella *Saintpaul infection.

## Case presentation

A 20-year-old previously healthy male presented to the emergency department with a one-day history of nausea, vomiting, severe abdominal pain, and profuse diarrhoea following recent travel to Thailand.

On initial assessment, he appeared clinically dehydrated, with dry mucous membranes, poor skin turgor, and sunken eyes. His vital signs revealed a temperature of 38°C, heart rate of 107 beats per minute, blood pressure of 100/70 mmHg, respiratory rate of 23 breaths per minute, and oxygen saturation of 98% on room air. Arterial blood gas analysis showed an elevated lactate level of 7 mmol/L (normal range 1-2 mmol/L). In view of a possible acute abdomen, the surgical team was initially involved. However, a computed tomography (CT) scan of the abdomen was unremarkable.

Notably, the patient tested positive for COVID-19 on admission, although his chest radiograph was normal. Given evidence of sepsis secondary to gastroenteritis in the context of COVID-19 infection, he was referred to the medical team and treated with empirical antibiotic therapy. Intravenous ceftriaxone (2 g once daily) and oral azithromycin (500 mg once daily) were initiated.

On day two of admission, despite appropriate antimicrobial coverage, the patient continued to have high-grade fevers (up to 39°C) and persistent tachycardia (heart rate approximately 160 beats per minute). Broad serological testing was performed to investigate a possible viral cause of fever, including HIV, Epstein-Barr virus (EBV), cytomegalovirus (CMV), malaria blood film, and hepatitis A and E serologies, all of which returned negative.

On day three, both blood and stool cultures returned positive for Salmonella species. Antimicrobial sensitivity testing revealed the isolate was sensitive to meropenem but resistant to ciprofloxacin, azithromycin, gentamicin, and piperacillin/tazobactam. Following microbiology consultation, the antibiotic regimen was escalated to intravenous meropenem (1 g every 8 hours) and vancomycin, with continuation of azithromycin (500 mg once daily). Despite escalation of treatment, the patient’s clinical status deteriorated further, with persistent tachycardia, recurrent pyrexia, ongoing gastrointestinal symptoms, and significantly raised inflammatory markers with C-reactive protein (CRP) of 400 mg/L (normal range <5 mg/L), along with electrolyte imbalances such as mild hyponatraemia and hypokalaemia (Table 1). 

**Table 1:** Key laboratory values during hospital admission WBC: white blood cell count, CRP: C-reactive protein.

Blood results	D1	D3	D4	D5	D6	D7	D8	D9	D10	Reference ranges
White cells (× 10^9^/L)	11.8	11.8	8.7	7.8	9	6.7	7.2	8.8	9.7	4.0–11.0
Neutrophils (× 10^9^/L)	8.5	8.8	6.6	4.8	5.3	3.9	3.4	4.3	5.4	2.0–7.5
CRP (mg/L)	18	263	X	479	440	287	137	170	216	<5
Sodium (mmol/L)	139	127	125	126	125	127	133	133	132	135–145
Potassium (mmol/L)	3.3	X	4.6	4.4	4.3	3.7	3.4	4.2	4.4	3.6–5.0

On day 7 after admission, an echocardiogram was performed to rule out infective endocarditis. Although it was negative for vegetations, it revealed severe global left ventricular systolic dysfunction and right ventricular impairment, consistent with tachycardia-induced cardiomyopathy. He was transferred to the coronary care unit (CCU) for close monitoring and telemetry, and initiation of full heart failure therapy.

**Figure 1:**
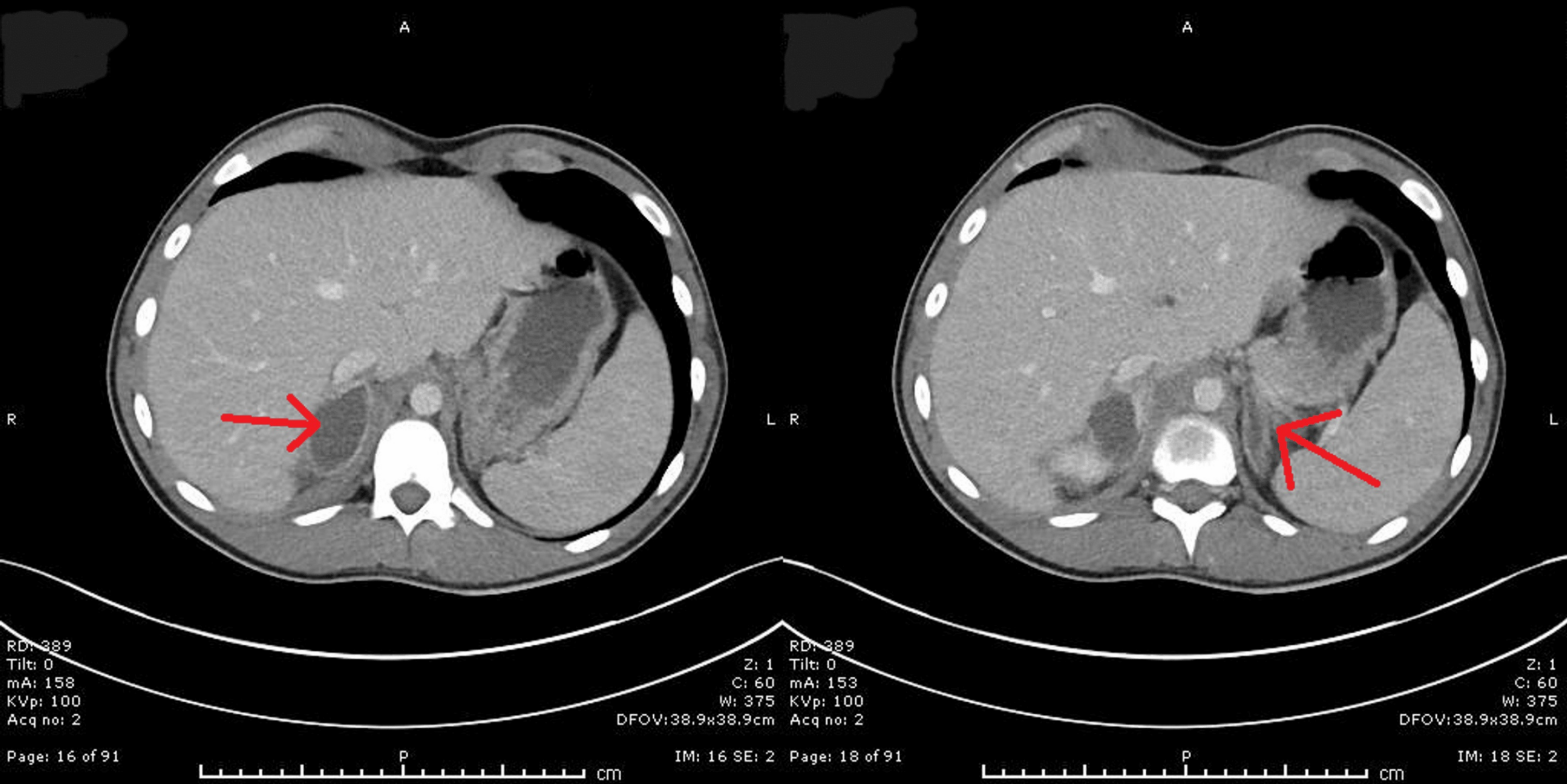
Contrast-enhanced CT abdomen demonstrating right adrenal abscess (left red arrow) and left adrenal abscess (right red arrow)

In an attempt to identify the source of persistent fever and symptoms, a repeat CT of the abdomen and pelvis was performed on day 10 of admission to rule out perforation or deep-seated collection. This revealed new bilateral adrenal abscesses, more prominent on the right side (Figure 1). The case was discussed with the regional infectious disease and endocrinology teams. A random cortisol level was 26 nmol/L, and he was also experiencing recurrent episodes of hypoglycaemia, prompting the initiation of high-dose corticosteroids alongside ongoing broad-spectrum antibiotics.

**Figure 2:**
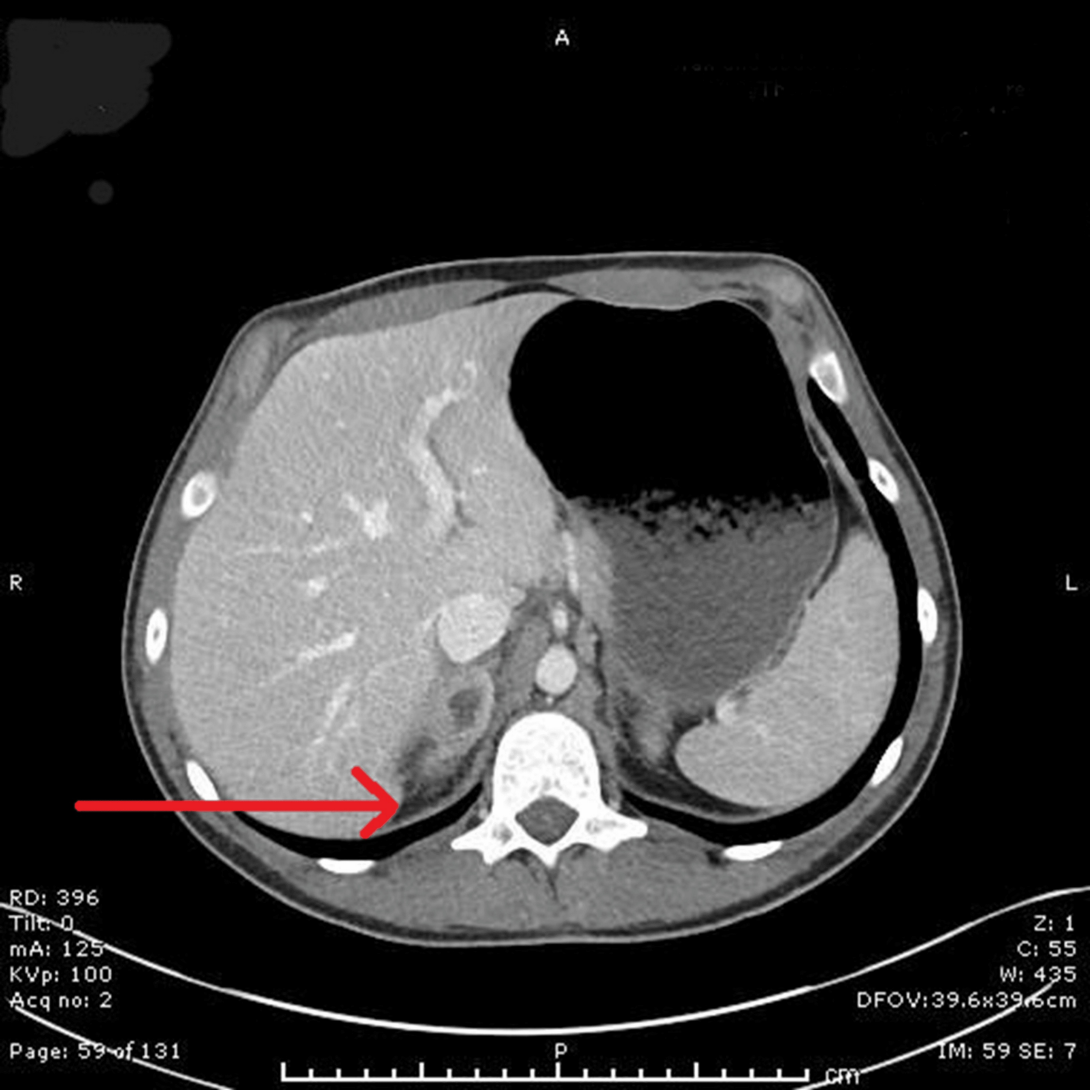
Contrast-enhanced CT (Day 38): Red arrow indicates reduced volume of right adrenal abscess; no evidence of recurrent left adrenal abscess

The patient was referred to a specialist infectious diseases unit, where he was managed conservatively, as the abscesses were not accessible for drainage by interventional radiology. He was treated with intravenous antibiotics and corticosteroids following multidisciplinary team discussion. He subsequently improved and was discharged home after two more weeks of antibiotics and a repeat CT scan, which showed improvement of the adrenal abscesses and normalisation of inflammatory markers (Figure 2).

**Figure 3:**
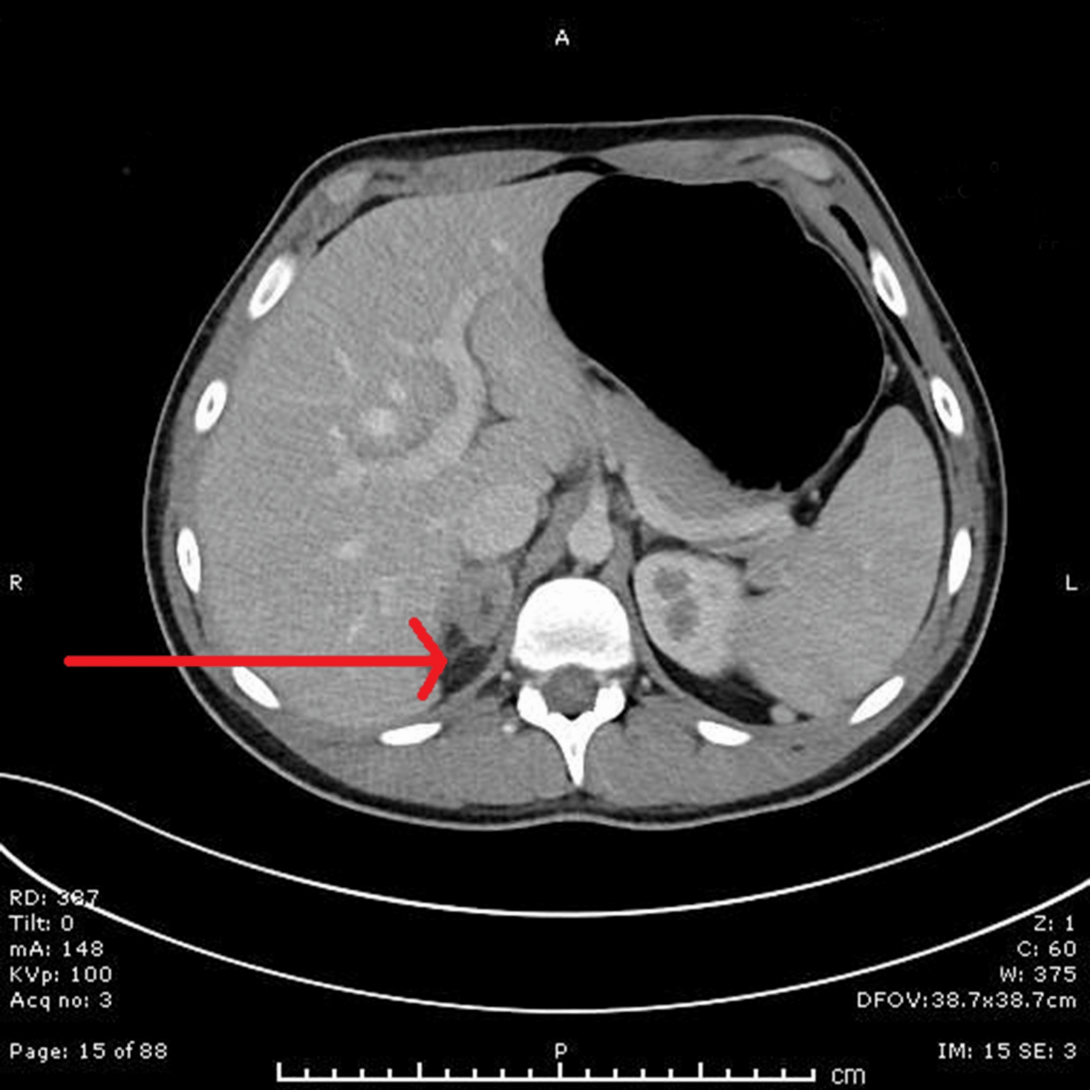
Contrast-enhanced CT (day 60): Further reduction in right adrenal abscess volume; left adrenal remains clear

Two weeks later, he re-presented with recurrent symptoms of nausea, vomiting, and lethargy. He was restarted on antibiotics and corticosteroids. A repeat CT scan demonstrated interval improvement in the adrenal abscesses (Figure 3). During the second admission, endocrine evaluation showed an elevated ACTH level of 273.1 ng/L and a low cortisol level of 26 nmol/L, confirming primary adrenal insufficiency. Renin was elevated at 8.3 and aldosterone was low at 61 pmol/L, consistent with mineralocorticoid deficiency. He was symptomatic at that time, with dizziness upon standing and postural drop, so he was started on fludrocortisone 100 micrograms daily. Thyroid function tests, serum testosterone, and HbA1c were all within normal limits, with no evidence of autoimmune polyglandular syndrome (Table 2).

**Table 2:** Hormonal panel demonstrating primary adrenal insufficiency ACTH: adrenocorticotropic hormone, TSH: thyroid-stimulating hormone.

Blood test	Result	Reference range
ACTH (ng/L)	273.1	7–63 ng/L
Cortisol (nmol/L)	26	138–690 nmol/L
Renin (mmol/L)	8.3	0.3–2.2 mmol/L
Aldosterone (pmol/L)	61	Up to 630 pmol/L
TSH (mU/L)	2.2	0.4–4.0 (mU/L)
Testosterone (nmol/L)	23.9	9.0–31 nmol/L

Before discharge, he was educated on sick-day rules and steroid replacement protocols. A follow-up plan was established, including monitoring of cortisol levels and a scheduled short Synacthen test. At the four-week follow-up after discharge, the patient reported feeling well and stable, and was taking his steroid replacement therapy regularly. His cortisol levels remained low both before and after the Synacthen test, and he was advised to continue his maintenance dose. An annual review was arranged for repeat imaging and reassessment of adrenal function.

## Discussion

This case highlights a rare but severe complication of *Salmonella *Saintpaul infection, leading to bilateral adrenal abscesses and adrenal insufficiency in an immunocompetent young adult. While *Salmonella *infections typically present as gastroenteritis, invasive forms can result in bacteraemia and abscess formation in various organs, including the adrenal glands [3-5]. *Salmonella *enteritidis is the most commonly reported serotype in clinical infections. In contrast, our case identified *Salmonella *Saintpaul through both blood and stool cultures, a serotype for which no prior infection outbreaks have been documented in Thailand. Notably, outbreaks of *Salmonella *Saintpaul have predominantly been reported in the United States and Brazil [6]. Our patient had recently travelled to Thailand, a region endemic for other *Salmonella *strains, suggesting a potentially under-recognised epidemiological presence or importation of this serotype. The initial symptoms of abdominal pain, vomiting, and diarrhoea were consistent with enterocolitis. However, persistent fever, elevated lactate of 4.5 mmol/L (normal range 0.6-2.4 mmol/L), and markedly raised C-reactive protein (CRP) of 457 mg/L (normal range <5 mg/L) suggested systemic involvement. Blood and stool cultures confirmed *Salmonella *Saintpaul, a rare serotype associated with travel-related outbreaks [7].

Persistent tachycardia led to tachycardia-induced cardiomyopathy, a reversible form of heart failure caused by sustained high heart rates. This condition is often underdiagnosed but can result in significant myocardial dysfunction. Studies show that early rate control and treatment of the underlying cause can lead to recovery of cardiac function [8-10]. 

Adrenal abscess formation is generally secondary to haematogenous dissemination from a distant infectious focus, with bilateral involvement more commonly observed in such cases. However, previously healthy individuals can also be affected, suggesting that certain *Salmonella *strains may have a predilection for adrenal tissue [3,4]. The adrenal glands’ unique vascular anatomy, with multiple arterial inputs but a single venous drainage, predisposes them to venous stasis ("damming"), haemorrhage, and microinfarction, providing a nidus for secondary bacterial infection [11].

*Salmonella *Saintpaul, a non-typhoidal serovar, can cause adrenal injury through systemic dissemination and immune-mediated mechanisms. During bacteraemia, the organism may seed adrenal tissue, where it hijacks programmed cell death pathways such as apoptosis, pyroptosis, necroptosis, and autophagy, resulting in cellular damage and inflammation [12]. Sepsis-induced adrenal insufficiency is a recognised phenomenon, often due to direct adrenal involvement or systemic inflammatory response impairing adrenal function. Studies indicate that relative adrenal insufficiency in sepsis is associated with poor outcomes, and early recognition is crucial for initiating corticosteroid therapy [13,14].

Individuals with any form of adrenal insufficiency are particularly vulnerable, as they cannot mount the normal cortisol surge needed during acute stressors. These stressors may include infection, especially sepsis, surgery, trauma, or significant emotional distress. Clinical presentation ranges from non-specific symptoms such as fatigue, nausea, and abdominal pain to progressive hypotension, hypoglycaemia, and electrolyte imbalance. Without prompt recognition and treatment, adrenal crisis can rapidly lead to circulatory collapse and death. In this case, recurrent hypoglycaemia and a critically low cortisol level (26 nmol/L) underscored the importance of early testing and therapeutic intervention with hydrocortisone replacement, which is lifesaving [15].

In terms of long-term management of hypoadrenalism, evidence from a systematic review suggests that nearly half (46.4%) of affected patients require sustained corticosteroid therapy, reflecting the risk of persistent adrenal insufficiency [16]. In this case, follow-up clinic assessment demonstrated consistently low serum cortisol levels, confirmed by a positive Synacthen test. Based on these findings, the patient was commenced on lifelong corticosteroid replacement therapy. This case underscores the importance of a multidisciplinary approach involving infectious disease specialists, endocrinologists, and the use of broad-spectrum antibiotics transitioning to targeted therapy as essential in controlling the infection. Additionally, patient education on steroid sick-day rules was crucial in preventing adrenal crisis following hospital discharge.

Learning points 

Persistent fever despite initial broad-spectrum antibiotic therapy should prompt reassessment for rare infectious foci, such as deep-seated abscesses or endovascular involvement, underscoring the need to consider adrenal pathology in febrile patients with persistent systemic symptoms and unexplained metabolic derangements. Additionally, tachycardia-induced cardiomyopathy is recognised as a reversible complication of systemic stress and infection, further highlighting the complexity of such cases. Effective management requires multidisciplinary care and comprehensive patient education to address both infectious and endocrine sequelae. This case also contributes to the limited literature on *Salmonella*-induced adrenal abscesses.

## Conclusions

This case illustrates a rare but clinically significant manifestation of *Salmonella *Saintpaul infection, culminating in bilateral adrenal abscesses and adrenal insufficiency in an immunocompetent individual. It underscores the importance of maintaining a high index of suspicion for extraintestinal complications in patients with persistent systemic symptoms following travel to endemic regions. Early recognition and multidisciplinary management, including targeted antimicrobial therapy, cardiac support, and hormone replacement, were pivotal to recovery. Clinicians should remain vigilant for adrenal involvement in systemic *Salmonella *infections, as timely intervention can be lifesaving and prevent long-term sequelae.
